# Production of Cinnamic and *p*-Hydroxycinnamic Acids in Engineered Microbes

**DOI:** 10.3389/fbioe.2015.00116

**Published:** 2015-08-20

**Authors:** Alejandra Vargas-Tah, Guillermo Gosset

**Affiliations:** ^1^Departamento de Ingeniería Celular y Biocatálisis, Instituto de Biotecnología, Universidad Nacional Autónoma de México, Cuernavaca, Mexico

**Keywords:** cinnamic acid, *p*-hydroxycinnamic acid, aromatics, metabolic engineering, phenylpropanoids, natural products, biotechnology

## Abstract

The aromatic compounds cinnamic and *p*-hydroxycinnamic acids (pHCAs) are phenylpropanoids having applications as precursors for the synthesis of thermoplastics, flavoring, cosmetic, and health products. These two aromatic acids can be obtained by chemical synthesis or extraction from plant tissues. However, both manufacturing processes have shortcomings, such as the generation of toxic subproducts or a low concentration in plant material. Alternative production methods are being developed to enable the biotechnological production of cinnamic and (pHCAs) by genetically engineering various microbial hosts, including *Escherichia coli*, *Saccharomyces cerevisiae*, *Pseudomonas putida*, and *Streptomyces lividans*. The natural capacity to synthesize these aromatic acids is not existent in these microbial species. Therefore, genetic modification have been performed that include the heterologous expression of genes encoding phenylalanine ammonia-lyase and tyrosine ammonia-lyase activities, which catalyze the conversion of l-phenylalanine (l-Phe) and l-tyrosine (l-Tyr) to cinnamic acid and (pHCA), respectively. Additional host modifications include the metabolic engineering to increase carbon flow from central metabolism to the l-Phe or l-Tyr biosynthetic pathways. These strategies include the expression of feedback insensitive mutant versions of enzymes from the aromatic pathways, as well as genetic modifications to central carbon metabolism to increase biosynthetic availability of precursors phosphoenolpyruvate and erythrose-4-phosphate. These efforts have been complemented with strain optimization for the utilization of raw material, including various simple carbon sources, as well as sugar polymers and sugar mixtures derived from plant biomass. A systems biology approach to production strains characterization has been limited so far and should yield important data for future strain improvement.

## Introduction

**Figure 1:**
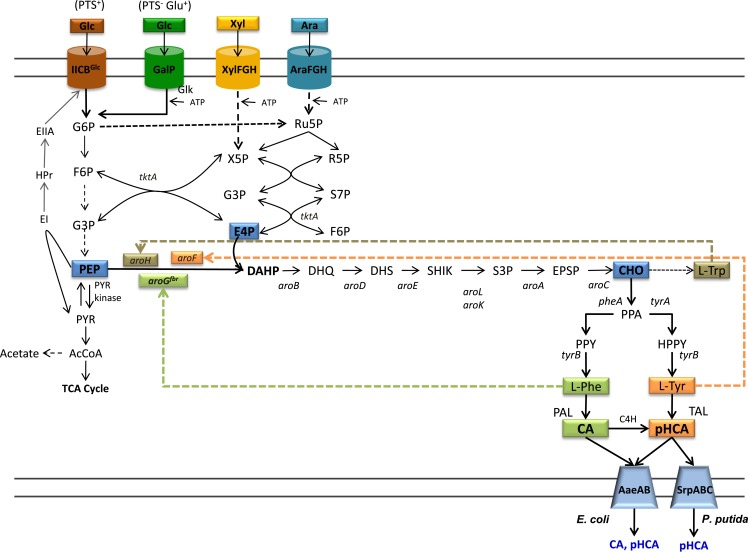
**Central metabolism, aromatics biosynthetic pathways, and transport pathways from engineered *E. coli***. Dashed arrows indicate multiple enzyme reactions. EI, PTS enzyme I; HPr, PTS phosphohistidine carrier protein; EIIA, PTS glucose-specific enzyme II; PTS IICB^Glc^, integral membrane glucose permease; GalP, galactose permease; XylFGH, xylose transport proteins, AraFGH, arabinose transport proteins; DAHPS, DAHP synthase; *aroG^fbr^*, gene encoding a feedback-inhibition-resistant version of DAHPS; *tktA*, transketolase; *tyrB*, tyrosine aminotransferase gene; PAL, phenylalanine ammonia lyase; TAL, tyrosine ammonia lyase; C4H, cinnamate 4-hydroxylase; AaeXAB, efflux pump from *E. coli*; SprABC, efflux pump from *P. putida*; G6P, glucose-6-phosphate; F6P, fructose-6-phosphate; G3P, glyceraldehyde-3-phosphate; PEP, phosphoenolpyruvate; R5P, ribose-5-phosphate; Ru5P, ribulose-5-phosphate; S7P, sedoheptulose-7-phosphate; X5P, xylulose-5-phosphate; PYR, pyruvate; AcCoA, acetyl-CoA; TCA, tricarboxylic acids.

Bacteria and plants have the natural capacity for synthesizing a large number of aromatic compounds from simple carbon sources. The shikimate or common aromatic pathway is the main central metabolic branch leading to several biosynthetic pathways that produce various aromatic metabolites (Figure 1). The aromatic amino acids l-phenylalanine (l-Phe), l-tyrosine (l-Tyr), and l-tryptophan (l-Trp) are primary metabolites synthesized from simple carbon sources by plants and bacteria. Secondary metabolites, such as phenylpropanoids, are derived from l-Phe and l-Tyr and are produced mainly by plants. The phenylpropanoid acids cinnamic acid (CA) and *p*-hydroxycinnamic acid (pHCA), also known as coumaric acid, are two metabolites having nutraceutical and pharmaceutical properties (Chemler and Koffas, 2008). They also have applications as precursors of chemical compounds and materials, such as high-performance thermoplastics (Kaneko et al., 2006; Sariaslani, 2007).

Both CA and pHCA are present in plant tissues at a low concentration. Therefore, complex procedures must be employed for their extraction and the yields are usually low. For these reasons, alternative production schemes are being explored. Several microbial species currently employed in biotechnological processes, possess part of the pathways required for CA and pHCA synthesis from simple carbon sources. Thus, by applying genetic engineering techniques to various microbial species, it has been possible to develop production strains with the novel capacity for synthesizing phenylpropanoid acids (Nijkamp et al., 2007; Vannelli et al., 2007a; Limem et al., 2008).

In this review, we focus on recent studies related to the application of genetic engineering strategies for the development of strains derived from *Escherichia coli*, *Pseudomonas putida*, *Streptomyces lividans*, and *Saccharomyces cerevisiae* for the production of the phenylpropanoid acids CA and pHCA. Production process development issues, such as product toxicity and carbon source utilization, are also discussed.

## The Shikimate Pathway and Derived Aromatic Biosynthetic Pathways

The common aromatic pathway or shikimate pathway includes common reactions leading to the specific biosynthetic pathways for l-Phe, l-Tyr, and l-Trp. Most of the reactions of the shikimate aromatic pathway are conserved among bacteria and plants; however, they can differ in terms of specific pathway regulation. The first reaction in the shikimate pathways is the condensation of central carbon metabolism intermediates phosphoenolpyruvate (PEP) and erythrose-4-phosphate (E4P) to yield 3-deoxy-d-*arabino*-heptulosonate-7-phosphate synthase (DAHP) (Figure 1). This reaction is catalyzed by the enzyme DAHP synthase. In bacteria, this step is usually regulated at the enzyme activity level by a feedback-inhibition allosteric mechanism on DAHP synthase. After six enzyme reactions, DAHP is converted to the intermediate chorismate (CHO). This compound is the metabolic branching point where pathways specific for the synthesis of l-Phe, l-Tyr, and l-Trp originate (Maeda and Dudareva, 2012).

The biosynthetic pathway for l-Tyr biosynthesis starts with the conversion of CHO to prephenate (PPA) by the enzyme chorismate mutase (CM). The intermediate PPA is converted to 4-hydroxyphenylpyruvate (HPP) in a reaction catalyzed by prephenate dehydrogenase (PDH). Finally, HPP is transformed to l-Tyr by transamination. Biosynthesis of l-Phe also starts with the conversion of CHO to PPA followed by a reaction yielding phenylpyruvate (PPY) in a reaction catalyzed by the enzyme prephenate dehydratase (PDT). It is common in bacteria to find bifunctional enzymes where the CM domain is fused to either the PDH or the PDT domain (Patel et al., 1977).

In most bacteria, l-Phe, l-Tyr, and l-Trp are the final products of their biosynthetic pathways. However, in plants and some bacteria, these amino acids are intermediates in pathways for the synthesis of secondary metabolites. The phenylpropanoids are a class of secondary metabolites produced by plants mainly as protection against biotic stress. These compounds also have medicinal use, such as antioxidants, UV screens, anticancer, antiviral, anti-inflammatory, anti-nitric oxide production, and antibacterial agents (Korkina et al., 2011). The first step in the phenylpropanoid pathway is the deamination of l-Phe to generate CA in a reaction catalyzed by the enzyme phenylalanine ammonia lyase (PAL). This compound is then transformed to pHCA by enzyme cinnamate 4-hydroxylase (C4H) (Achnine et al., 2004). pHCA is a precursor for a large number of metabolites including flavonoids and lignans. These compounds have important structural and protective functions in plants (Emiliani et al., 2009). Several of the characterized PAL enzymes can also employ l-Tyr as a substrate, thus displaying tyrosine ammonia lyase (TAL) activity and producing pHCA directly from l-Tyr (Cochrane et al., 2004; Cui et al., 2014). Therefore, the term PAL/TAL is usually employed in naming this type of enzymes. At present, known TAL enzymes have substrate specificity toward both l-Phe and l-Tyr. The specificity to l-Tyr of the TAL from *Rhodobacter sphaeroides* has been modified to l-Phe by changing amino acid residue His89 that is conserved in enzymes with TAL activity to Phe. This result suggests that it might be possible to modify the substrate specificity of enzymes that display PAL activity by employing protein engineering approaches (Louie et al., 2006).

## Engineering of Microbes for Production of Phenylpropanoid Acids

Microbial species traditionally employed in biotechnological processes do not have the natural capacity for synthesizing phenylpropanoid acids. Therefore, genetic modifications are required to generate CA or pHCA production strains. Since enzymes having PAL/TAL activities are not present in industrial microbial strains, a key modification to generate phenylpropanoid acids productions strains is the heterologous expression of genes encoding these proteins. These strains are usually further engineered by modifying carbon flow distribution in central and biosynthetic pathways with the aim of increasing l-Phe and l-Tyr synthesis capacity. Such strategies can be complemented with the engineering of substrate utilization and product tolerance. These strategies are presented and discussed in the following sections.

## Engineering of Key Pathways/Targets for the Production of CA and pHCA

### PAL/TAL enzymes as key reactions

The heterologous expression of genes encoding PAL/TAL activities is an essential modification to generate CA and pHCA production strains. Genes encoding PAL/TAL enzymes have been expressed in various microbial hosts to enable the capacity of transforming l-Phe and l-Tyr into CA and pHCA, respectively. Table 1 shows the sources of various PAL/TAL enzymes and their kinetic parameters (when known). As it can be observed, the genes employed originate from various biological groups, including bacteria, yeasts, and plants. The kinetic characterization of these enzymes provides data that can be used to compare them regarding substrate affinity and catalytic efficiency. Several enzymes of this family display both PAL and TAL activities. However, a wide range of *K*_m_ values can be observed for substrates l-Phe and l-Tyr (Table 1). A microbial strain that expresses a gene encoding a PAL/TAL enzyme acquires the capacity for transforming l-Phe or l-Tyr into the corresponding phenylpropanoid acid. Therefore, in a production context, the aromatic amino acid must be supplemented to the culture medium, where it is then internalized and deaminated by the PAL/TAL enzyme.

**Table 1:** **Kinetic parameters for phenylalanine ammonia lyase and tyrosine ammonia lyase enzymes from various organisms**.

Organism	*K*_m_ l-Phe (μM)	*K*_m_ l-Tyr (μM)	Reference
*Rhodobacter capsulatus*	1277	15.6	Kyndt et al. (2002)
*Zea mays*	270	19	Rosler et al. (1997)
*Petroselinum crispum*	24.5	7.8	Appert et al. (1994)
*Rhodobacter sphaeroides*	560	60	Vannelli et al. (2007b)
*Phanerochaete chrysosporium*	161	44	Xue et al. (2007)
*Trichosporon cutaneum*	2330	432	Vannelli et al. (2007a)
*Rhodotorula minuta*	584	212	Vannelli et al. (2007a)
*Rhodotorula graminis*	448	154	Vannelli et al. (2007a)
*Streptomyces maritimus* (*encP*)	23	–	Xiang and Moore (2005)
*Rhodotorula glutinis*	1340	560	Zhu et al. (2013)
*Arabidopsis thaliana* (*PAL1*)	68	–	Cochrane et al. (2004)
*Arabidopsis thaliana* (*PAL2*)	64	–	Cochrane et al. (2004)
*Arabidopsis thaliana* (*PAL3*)	256	–	Cochrane et al. (2004)
*Arabidopsis thaliana* (*PAL4*)	71	–	Cochrane et al. (2004)
*Saccharothrix espanaensis*	2860	15.5	Berner et al. (2006)

### Engineering of pathway regulation

Most microbial hosts have the metabolic capacity for synthesizing l-Phe of l-Tyr from simple carbon sources. Therefore, to reduce production costs, it is desirable to enhance endogenous biosynthesis of the aromatic substrates to avoid having to add them to the culture medium. The strategies for generating l-Phe or l-Tyr overproducer strains are well-known and they have been applied to generate microbial strains that can produce these amino acids at grams level from simple carbon sources (Ikeda and Katsumata, 1992; Ikeda et al., 2006; Báez-Viveros et al., 2007; Lütke-Eversloh and Stephanopoulos, 2007, 2008; Chávez-Bejar et al., 2008; Juminaga et al., 2012; Kang et al., 2012). The elimination of enzyme feedback inhibition regulation and transcriptional regulatory processes are the most common modification that enhances flux to the l-Phe of l-Tyr biosynthetic pathway. The high-level expression of feedback inhibition resistant (fbr) versions of enzyme DAHP synthase causes an increase in carbon flow from central metabolism to the shikimate pathway. This increased flux toward CHO synthesis can be redirected to the specific l-Phe and l-Tyr biosynthetic pathway by overexpressing fbr versions of PDT and PDH, respectively. In addition to the aforementioned strategies for increasing synthesis capacity of aromatic amino acids, other approaches, such as a combination of strain random mutagenesis and selection, have proven successful for generating mutants that overproduce l-Phe of l-Tyr (Bongaerts et al., 2001). Random mutagenesis and selection schemes have also been employed to generate *P. putida* strains for the production of CA and pHCA. The *P. putida* strain S12 was isolated in cultures containing a high styrene concentration and has been shown to be solvent tolerant (Weber et al., 1993). This strain was engineered by expressing the gene coding for the PAL/TAL from *R. toruloides*. To increase l-Phe biosynthesis capacity, this strain was subjected to random mutagenesis and a selection process on the toxic analog m-fluoro-phenylalanine. An isolated mutant produced 5 mM CA from glucose (Nijkamp et al., 2005). In addition to CA, this strain produced a very low amount of pHCA. To obtain a high-level pHCA producer strain, random mutagenesis and m-fluoro-phenylalanine selection were employed, considering that l-Phe and l-Tyr share steps in their biosynthetic pathways. Following this procedure, a strain was obtained that showed a 14-fold increase in pHCA synthesis. However, degradation of pHCA was observed in the culture. It is known that *P. putida* has a pCHA catabolic pathway. The gene *fcs* encoding feruloyl-CoA synthetase was inactivated in *P. putida* S12, thus eliminating the first step in the degradative pathway. This modification caused a 2.5-fold increase in pHCA titer (224 μM); however, a large amount of CA was also produced (350 μM). To reduce CA production, l-Phe auxotrophic mutants were generated by random mutagenesis. One of such mutants produced 860 and 70 μM of pHCA and CA, respectively (Nijkamp et al., 2007). These results demonstrate how random mutagenesis schemes coupled to selection with toxic analogs can be employed to successfully yield overproducing strains. However, a drawback of such methods is the lack of knowledge on the specific mutations responsible for the observed phenotype. The application of genome sequencing to characterize such mutants should yield information that will allow for the future rational design of production strains.

### Engineering building blocks supply

The capacity for synthesizing aromatic amino acids can be improved by increasing availability of central metabolism precursors PEP and E4P. The enzymes transketolase (Tkt) and transaldolase (Tal) from the pentose phosphate pathway participate in E4P metabolism. The overexpression of each enzyme showed a positive effect on DAHP synthesis from glucose. It was found that *tktA* overexpression had a larger positive effect on increasing E4P availability for aromatics biosynthesis. Unexpectedly, simultaneous expression of Tkt and Tal genes did not show a synergistic effect on DAHP synthesis from glucose (Draths et al., 1992; Lu and Liao, 1997). PEP participates in several anabolic and catabolic pathways. In addition, it serves as phosphate donor during import and phosphorylation of glucose and other sugars by the PEP:sugar phosphotransferase system (PTS) (Erni, 2012). When a bacterium grows on a sugar source that can be internalized by the PTS, such as glucose, this system is the major consumer of PEP as one mole of this molecule is required to transport and phosphorylate 1 mole of glucose, generating glucose-6-phosphate (G6P), and PYR (Figure 1). Various studies have stabilized that PEP availability determines the yield of aromatic amino acids synthesized from glucose (Patnaik et al., 1995). The maximal theoretical yield for the synthesis of aromatics precursor DAHP from glucose in a strain with an active PTS is 0.43 mol/mol. However, if glucose phosphorylation is PEP independent, the maximal theoretical yield could be increased twofold to 0.86 mol/mol (Báez et al., 2001). The reactions catalyzed by enzymes Ppc and Pyk consume PEP and for this reason they have become targets for strain improvement. The inactivation of gene *ppc* caused a 10-fold increase in l-Phe production, but μ was severely affected (Miller et al., 1987). The enzyme Pps, encoded by *ppsA*, catalyzes a gluconeogenic reaction synthesizing PEP from PYR. The overexpression of *ppsA* in *E. coli* has been shown to increase DAHP production to near the maximum theoretical yield. However, it was also determined that *ppsA* overexpression caused partial growth inhibition (Patnaik et al., 1995).

### Engineering of substrate utilization

To increase PEP availability for aromatics production, one approach involves inactivation of PTS activity and its replacement by alternate import and phosphorylation mechanisms. Mutant strains of *E. coli* lacking PTS activity (PTS^−^) have been generated and characterized. These PTS^−^ mutants display very low rates of glucose consumption and growth (PTS^−^ glucose^−^ phenotype). Therefore, they are not useful for production applications. For this reason, several strategies have been followed to improve glucose import capacity in these mutant strains. Starting from a PTS^−^ glucose^−^ strain, a continuous culture system was employed to select evolved bacterial clones displaying a fourfold higher specific growth rate (μ) (PTS^−^ glucose^+^ phenotype) (Flores et al., 1996). The characterization of these laboratory evolved strains revealed that glucose uptake and phosphorylation are carried out by a PTS-independent mechanism involving galactose permease (GalP) and glucokinase (Glk) (Flores et al., 1996, 2002). The enzyme Glk phosphorylates the cytoplasmic glucose employing ATP as the phosphate donor, so in these strains, PEP is not consumed for this reaction. An alternative method for generating PTS^−^ glucose^+^ strains involves the overexpression of genes encoding native or heterologous proteins having glucose import and ATP-dependent phosphorylating activities (Snoep et al., 1994; Hernández-Montalvo and Martínez, 2003). The characterization of several PTS^−^ glucose^+^ strains shows that aromatics yield from glucose can be increased to a level close to the maximal theoretical yield calculated for a strain with PEP-independent glucose import (Flores et al., 1996; Báez et al., 2001).

The use of substrates derived from lignocellulosic hydrolyzates is a current trend in the development of biotechnological processes. These are abundant and relatively inexpensive carbon sources that can become the basis of sustainable production processes. Although the composition of such materials differ according to their origin, most of them contain a mixture of pentoses and hexoses, mainly glucose, arabinose, and xylose. The efficient utilization of such sugar mixture by the production strains is an important characteristic. Therefore, microbial species that naturally consume such mixtures must be employed, or genetic modification must be employed to provide such trait. The PTS is involved in carbon catabolic repression, a regulatory process responsible for the sequential utilization of mixtures of carbon sources in *E. coli* and other bacteria. To determine the effect of PTS inactivation on sugar mixtures utilization and aromatics acids production, a combinatorial study was reported where the effect of various phenotypes on CA and pHCA production were compared. The authors generated strains derived from wild type and a PTS^−^ glucose^+^ mutant that expressed PAL/TAL from *Rhodotorula glutinis* or *A. thaliana* as well as genes encoding an fbr version DAHP synthase and Tkt. These strains were grown in medium supplemented with glucose, arabinose, xylose, or a simulated lignocellulosic hydrolyzate containing a mixture of these three sugars and acetate. When grown in the simulated lignocellulosic hydrolyzate, sequential sugar utilization was observed in the wild-type strain, whereas they were simultaneously consumed by the PTS^−^ glucose^+^ strain. This is a trait that might prove to increase productivity when employing hydrolyzates from lignocellulosic raw materials (Vargas-Tah et al., 2015).

The organism *S. lividans* has the natural capacity to grow employing various complex carbon sources, including cello-oligosaccharide and xylo-oligosaccharide (Noda et al., 2012). This is a useful trait since no previous physical or chemical treatment of the lignocellulosic biomass is required to generate free sugars. This bacterium was modified for CA production by the heterologous expression of gene *encP* coding for a PAL from *Streptomyces maritimus*. Production of CA was observed when culturing in complex medium supplemented with glucose or glycerol as carbon source. Carbon sources that can be derived from biomass were also tested, including xylose, xylan, and raw starch with CA titers of 300, 130, and 460 mg/L, respectively (Noda et al., 2011). In another report, the *S. lividans* strain expressing *encP* was employed for producing CA from oligosaccharides as carbon sources in complex medium. This strain produced 490, 400, and 160 mg/L of CA with cello-oligosaccharide, xylo-oligosaccharide, and Avicel as carbon sources, respectively (Noda et al., 2012). A strain of *S. lividans* was constructed for pHCA production by expressing a gene encoding a TAL from *Rhodobacter sphaeroides*. This strain produced 786 and 736 mg/L of pHCA from glucose or cellobiose as carbon source. This strain was further modified by expressing a gene encoding an endoglucanase from *Thermobifida fusca* YX. The recombinant *S. lividans* strain produced 500 mg/L of pHCA from phosphoric acid swollen cellulose (Kawai et al., 2013).

## Engineering of Product Tolerance

It is known that CA and pHCA are toxic compounds for several microorganisms (Qi et al., 2007; Sariaslani, 2007; Vargas-Tah et al., 2015). For example, a concentration of 10 g/L of pHCA completely abolishes growth in *E. coli* (Sariaslani, 2007). Under production conditions where these compounds accumulate to high titers, a negative effect on productivity and cell viability would be expected. Two general approaches have been followed to mitigate the negative effects of CA and pHCA accumulation on production strain performance. The first one is based on the overexpression of efflux systems that can employ pHCA as substrate. In one study, it was demonstrated that *E. coli* mutants in TolC, the outer membrane factor for several efflux systems, are more sensitive to the negative effects of pHCA. The main multidrug efflux system in *E. coli* is AcrAB, it requires TolC to export several kinds of toxic compounds. An increase in sensitivity to pHCA of *acrAB* mutants showed that this compound is a substrate of this efflux system. However, the sensitivity was higher in a strain with mutated efflux system and TolC, suggesting that other(s) efflux system(s) could be active with pHCA. As a strategy to identify gene candidates encoding proteins involved in pHCA efflux, transcriptome analysis was performed with *E. coli* grown in the presence of this aromatic acid. This study found genes *aaeA* and *aaeB* to be upregulated. Genes *aaeXAB* encode an efflux pump, when this operon was overexpressed in *E. coli*, a twofold increase in tolerance to pHCA was observed (Figure 1) (Van Dyk et al., 2004; Sariaslani, 2007). This study also found that AaeXAB is functional in the absence of TolC, thus indicating the existence of an additional aromatics acids efflux system that is dependent on TolC (Van Dyk et al., 2004).

The second approach to avoid the toxic effects or aromatic acid is based on the use as production host of an organism having a natural high tolerance to these compounds. The *P. putida* strain S12 was isolated from cultures containing a high concentration of styrene. Characterization of this strain has revealed that it is tolerant to various organic solvents (Weber et al., 1993). An efflux pump encoded by genes *srpABC* in this strain has been identified as an important factor in solvent tolerance and in its capacity to export chemical products (Figure 1). Their expression in a solvent-sensitive *P. putida* strain increases its resistance to solvents (Kieboom et al., 1998). As mentioned above, engineering of this strain has led to production hosts displaying the highest reported CA and pHCA titers in production cultures (Nijkamp et al., 2005, 2007).

## Microbial Hosts Employed for Phenylpropanoid Acids Production

As mentioned above, various microbial strains have been modified for the production of CA or pHCA by employing diverse metabolic engineering strategies. This includes Gram-negative, Gram-positive, and eukaryotic organisms. The Gram-negative bacterium *E. coli* is a facultative anaerobe that can employ a large variety of organic compounds as carbon and energy sources. *E. coli* was the first organism modified by genetic engineering and it is currently employed as an important model in metabolic engineering experiments. The existence of a wide array of genetic modification techniques developed for *E. coli* has enabled the engineering of this organism for aromatic acids production. As shown in Table 2, CA and pHCA *E. coli* production strains have been generated that express PAL/TAL enzymes from various origins, having the capacity to employ single sugars or mixtures as substrates. Even though *E. coli* is a useful host organism, increasing its tolerance to aromatic acids is still a challenge for developing robust production strains.

**Table 2:** **Comparison of production parameters for aromatic acids synthesized by engineered microbial strains**.

Organism	PAL/TAL	Carbon source	pHCA (mg/L)	CA (mg/L)	Reference
*E. coli* (w3110)	*R. glutinis*	Glucose	7.0	4.9	Vannelli et al. (2007a)
*E. coli* (DH10B)	*R. glutinis*	Glucose	46.0	41.5	Vannelli et al. (2007a)
*E. coli* (ATCC 31884)	*R. glutinis*	Glucose	24.6	56.3	Vannelli et al. (2007a)
*E. coli* (BL21*AI)	*T. cutaneum*	LB	79	186	Vannelli et al. (2007a)
*E. coli* (PTS^−^)	*R. glutinis*	Glucose	15.0	29.0	Vargas-Tah et al. (2015)
*E. coli* (PTS^−^)	*A. thaliana*	Glucose	–	78.5	Vargas-Tah et al. (2015)
*E. coli* (W3110)	*A. thaliana*	Arabinose	–	151.4	Vargas-Tah et al. (2015)
*E. coli* (W3110, *pheA*^−^)	*R. glutinis*	Simulated hydrolyzate	58.4	15.6	Vargas-Tah et al. (2015)
*E. coli* (W3110)	*A. thaliana*	Simulated hydrolyzate	–	55.9	Vargas-Tah et al. (2015)
*S. cerevisiae*	*R. glutinis*	Glucose	2.3	–	Vannelli et al. (2007a)
*S. cerevisiae*	*R. glutinis*	Raffinose	31.8	–	Vannelli et al. (2007a)
*P. putida* S12 C1	*R. toruloides*	Glucose	36.8	46.5	Nijkamp et al. (2007)
*P. putida* S12 C3	*R. toruloides*	Glucose	141.2	10.4	Nijkamp et al. (2007)
*P. putida* S12 C3	*R. toruloides*	Glucose	1740.1	22.2	Nijkamp et al. (2007)
*P. putida* S12	*R. toruloides*	Glucose	–	61.4	Nijkamp et al. (2005)
*P. putida* S12	*R. toruloides*	Glucose	–	740.3	Nijkamp et al. (2005)
*P. putida* S12	*R. toruloides*	Glycerol	–	799.5	Nijkamp et al. (2005)
*S. lividans*	*R. sphaeroides*	Glucose	786	–	Kawai et al. (2013)
*S. lividans*	*R. sphaeroides*	Cellobiose	736	–	Kawai et al. (2013)
*S. lividans*, endoglucanase expressing	*R. sphaeroides*	Glucose	753	–	Kawai et al. (2013)
*S. lividans*, endoglucanase expressing	*R. sphaeroides*	Cellobiose	743		Kawai et al. (2013)
*S. lividans*, endoglucanase expressing	*R. sphaeroides*	Cellulose	500	–	Kawai et al. (2013)
*S. lividans*	*S. maritimus*	Glucose	–	80.0	Noda et al. (2012)
*S. lividans*	*S. maritimus*	Glucose	–	209.8	Noda et al. (2012)
*S. lividans*	*S. maritimus*	Glycerol	–	450	Noda et al. (2011)
*S. lividans*	*S. maritimus*	Xylose	–	300.0	Noda et al. (2012)
*S. lividans*	*S. maritimus*	Xylan	–	130.0	Noda et al. (2011)
*S. lividans*	*S. maritimus*	Raw starch	–	460.0	Noda et al. (2011)
*S. lividans*	*S. maritimus*	Cello-oligosaccharide	–	490	Noda et al. (2012)

The genus *Streptomyces* includes Gram-positive organisms displaying the natural capacity for synthesizing antibiotics and other secondary metabolites. Among them, the bacterium *S. lividans* synthesizes enzymes that enable it to consume various biomass-derived polymers, such as xylan, starch, cello-oligosaccharide, and xylo-oligosaccharide (Noda et al., 2012). This organism has been genetically modified for production of CA and pHCA from simple sugars or polymers as carbon sources. The rate of production for the aromatic acids was lower with sugar polymers when compared to simple sugars. This indicates that further strain improvement would be required to increase the cellular activities related to polymer consumption (Table 2) (Noda et al., 2011, 2012; Kawai et al., 2013).

The Gram-negative bacterium *P. putida* is a versatile organism, displaying the capacity to colonize diverse niches. A *P. putida* strain designated S12 was isolated from cultures containing a high styrene concentration and it has been shown to be tolerant to several organic solvents (Weber et al., 1993). *P. putida* S12 has been engineered to generate CA and pHCA production strains. These strains have reached the highest aromatic acids titers in production cultures a result likely attributed to the solvent tolerance of the progenitor strain (Table 2). The introduction of functions related to sugar polymer consumption would bring *P. putida* S12 closer to an ideal aromatics acids production strain.

The yeast *S. cerevisiae* is a unicellular eukaryotic organism that has been employed as an important biological model. In addition, yeast is an industrial organism that has been fundamental to the development of various fermentative processes. Currently, there is one example of the genetic modification of *S. cerevisiae* for pHCA production. A production strain was generated by expressing the *R. glutinis* PAL/TAL. This *S. cerevisiae* strain also expressed the Cytochrome *P*-450 enzyme system from the plant *Helianthus tuberosus*. When culturing this yeast strain with glucose or raffinose as carbon sources, the highest titers of pHCA were 14.6 and 202.8 (Vannelli et al., 2007a,b).

## Production of CA and pHCA Using Biotransformation

As mentioned above, biological synthesis of CA and pHCA starting from simple or complex sugars has the potential for becoming a relatively inexpensive manufacturing scheme. However, such processes must deal with the issue of product toxicity, which places an upper limit on attainable product titers. A solution to this problem would be to employ a manufacturing alternative based on performing a biotransformation where l-Phe and l-Tyr are employed as substrates for the production of CA and pHCA, respectively. In this scheme, the cell host is required only for the synthesis of the PAL/TAL enzyme, which can be purified for performing the deamination reactions. Production of pHCA was studied by comparing PAL/TAL enzymes from yeast *R. glutinis* (*Rg*TAL) and fungus *Phanerochaete chrysosporium* (*Pc*TAL). The genes coding for these enzymes were expressed in *E. coli* and the protein products were purified. Characterization of purified enzymes showed that *Pc*TAL is thermostable, with a maximal activity at 55–60°C. In experiments with whole cells of *E. coli* expressing *Pc*TAL, 42.2 g/L of pHCA were produced with a specific productivity of 1.11 g/g h (Xue et al., 2007). In another report, the *Rg*TAL was stabilized by encapsulation within polyethyleneimine-mediated biomimetic silica. The free enzyme lost all its activity when exposed 1 h to a temperature of 60°C, whereas encapsulated *Rg*TAL retained 43% of its initial activity (Cui et al., 2015). These results show how a natural version of PAL/TAL or one that has been stabilized can constitute viable alternatives for the synthesis of aromatic acids from l-Phe or l-Tyr substrates.

## Conclusion and Outlook

The development of microbial strains for the production of CA and pHCA from simple carbon sources involves extensive engineering of cellular metabolism. In addition to improving production characteristics, these modifications will likely also cause unexpected alterations to the cell’s physiology. System biology approaches offer the opportunity of better understanding the consequences of genetic modifications and the responses to various stress factors during the production stage. The application of omics-based approaches, such as transcriptomics, proteomics, fluxomics, and metabolomics, provides a comprehensive view of the cell’s physiology response to various genetic modifications as well as environment factors, such as product toxicity. For the case of microbial strains for the production of CA and pHCA, there is a lack of studies based on omics approaches. However, there are some reports focusing on the study of strains modified for the production of precursors of aromatic acids. In one report, proteome analysis was performed to understand the effect of inactivating a PYR kinase PykF in *E. coli*. Among proteins differentially expressed in the mutant strain, some were related to E4P synthesis and the common aromatic pathway, suggesting a higher capacity for aromatics synthesis (Prabhakar et al., 2007). Transcriptome analysis was performed to compare a PTS^+^ and a PTS^−^ glucose^+^ *E. coli* strain modified for l-Phe production. Among differentially expressed genes, it was found that operon *acs*-*actP* that is involved in acetate consumption was upregulated in the PTS^−^ glucose^+^ strain (Báez-Viveros et al., 2007). This response is consistent with the lower level of acetate accumulation in culture medium observed for strain PTS^−^ glucose^+^ when compared to PTS^+^. These results provide useful data that helps in identifying genetic targets for strain improvement. Future studies focused on characterizing CA and pHCA production strains will likely identify novel targets for strain optimization.

Aromatic acids CA and pHCA are valuable chemicals having direct applications and serving also as precursors for the synthesis of a large number of useful compounds. During the last years, various microbial hosts have been modified by metabolic engineering to generate production strains. These efforts have been fundamental for defining strain development strategies and for identifying factors that limit productivity. In contrast to other biotechnological products where a single microbial host is usually employed, for the case of CA and pHCA production, several different species show promise as production platforms. As reviewed here, *E. coli*, *S. cerevisiae*, *P. putida*, and *S. lividans* display particular characteristics that can favor aromatics acids production. Although much progress has been made with regard to production strain construction and process development, the yields of aromatic acids are still low when compared to other aromatic products (Bongaerts et al., 2001). An important factor limiting productivity is the toxicity of CA and pHCA. In this regard, studies identifying genes encoding efflux systems in *E. coli* and *P. putida* S12 enable a better understanding of the processes involved in mitigating aromatic acids toxicity (Kieboom et al., 1998; Van Dyk et al., 2004). The overexpression of these genes in each organism clearly increases resistance to toxic compounds. It remains to be determined if the solvent-tolerance trait can be transferred to a different species. The use of an omics approach to determine the transcriptional response to CA and pHCA should prove to be valuable for identifying systems that participate in toxic resistance in other microbial species.

Generating a single product is usually the expected outcome in a biotechnological production system. In microbial strains engineered to produce aromatic acids from simple carbon sources, it has been shown that synthesis of CA as only product is possible, as a result of PAK specificity toward l-Phe. However, this is not the case for pHCA, since known TAL enzymes can also employ l-Phe as substrate. Therefore, pHCA is produced always with a certain amount of CA. Although downstream processing could be employed to separate pHCA from CA, this approach would result in increased production costs. Another solution to this issue could be based on applying protein engineering methods to modify substrate specificity of a TAL enzyme for reducing or abolishing CA production, while maintaining high-catalytic activity to increase production of pHCA. As an alternative, the search for novel TAL proteins in natural diversity has the potential for finding enzymes having substrate specificity only toward l-Tyr.

**Figure 2:**
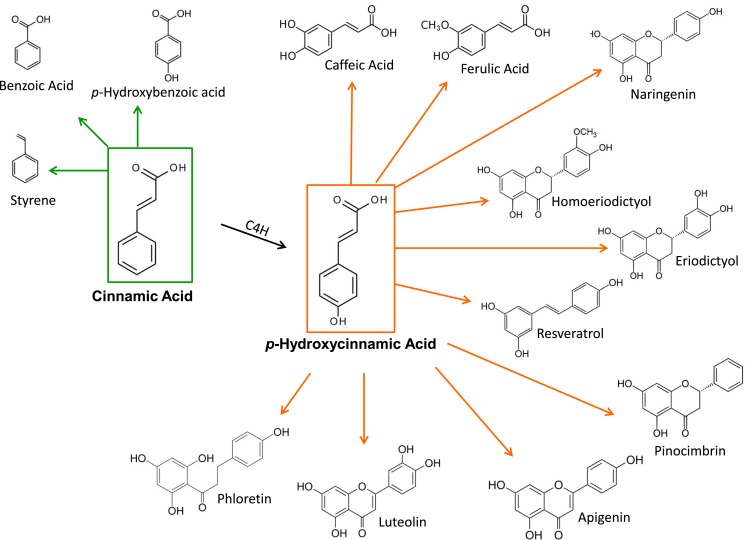
**Plant metabolites produced from CA and pHCA by engineered microbial strains**.

Microbial strains having the capacity for producing CA or pHCA have been employed as platforms for the synthesis of various phenylpropanoid compounds. These include simple phenylpropanoids as well as lignoids, flavonoids, coumarins, and other related compounds (Figure 2) (Dixon and Steele, 1999). These plant metabolites have been shown to have pharmacological activities, such as antioxidants, anticancer, antiviral, anti-inflammatory, anti-nitric oxide production and antibacterial agents, among others (Dhanalakshmi et al., 2002). The microbial production of these compounds represents an attractive alternative to plant tissue extraction processes. However, at present, these microbial strains produce a low level of these plant compounds. It can be expected that some of the metabolic engineering strategies applied to CA and pHCA production strains, as reviewed here, should provide a basis for the future improvement of microbial strains that synthesize useful plant metabolites.

## Conflict of Interest Statement

The authors declare that the research was conducted in the absence of any commercial or financial relationships that could be construed as a potential conflict of interest.
